# Soluble N-cadherin: A novel inhibitor of VSMC proliferation and intimal thickening

**DOI:** 10.1016/j.vph.2015.11.040

**Published:** 2016-03

**Authors:** Cressida A. Lyon, Kerry S. Wadey, Sarah J. George

**Affiliations:** School of Clinical Sciences, Research Floor Level 7, Bristol Royal Infirmary, Upper Maudlin St, Bristol BS2 8HW, UK

**Keywords:** Proliferation, Intimal thickening, Smooth muscle, Endothelial, Cadherin

## Abstract

Reoccurrence of symptoms occurs in 30–50% of coronary artery disease patients receiving vein grafts or bare-metal stents due to intimal thickening (restenosis). Restenosis is caused by vascular smooth muscle cell (VSMC) migration and proliferation. New therapeutic approaches that reduce VSMC migration and proliferation while promoting endothelial cell (EC) coverage are required. We assessed the effect of a soluble form of N-cadherin (SNC-Fc, a fusion of the extracellular portion of N-Cadherin to a mutated Fc fragment of IgG), a cell–cell junction molecule, on human saphenous VSMC proliferation and migration in vitro. We also assessed its effect on intimal thickening in a validated human ex vivo organ culture model. We observed that SNC-Fc significantly inhibited VSMC proliferation and to a lesser extent migration. The anti-proliferative effect of SNC-Fc was mediated by the interaction of SNC-Fc with the FGFR, rather than through inhibition of β-catenin signalling. SNC-Fc also significantly reduced intimal thickening by ~ 85% in the ex vivo organ culture model. SNC-Fc treatment inhibited proliferation of the intimal cells but did not affect migration. SNC-Fc reduced EC apoptosis, without detrimental effects on EC proliferation and migration in vitro. Importantly SNC-Fc increased EC coverage in the ex vivo model of intimal thickening. In conclusion, we suggest that SNC-Fc may have potential as an anti-proliferative therapeutic agent for reducing restenosis which has no detrimental effects on endothelial cells.

## Graphical abstract

**Figure:**
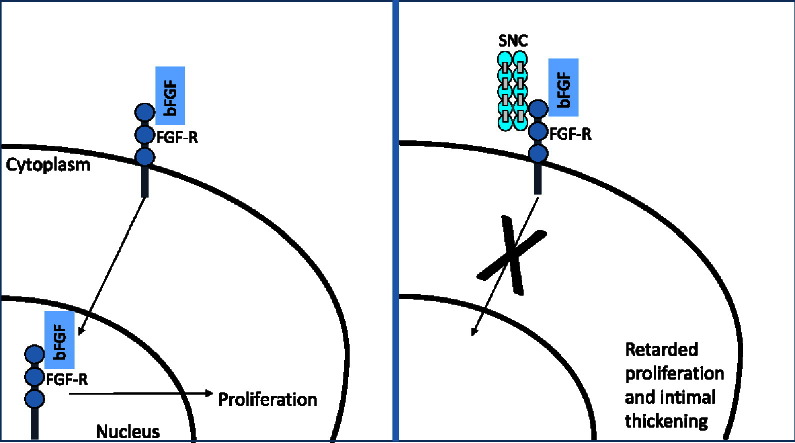

## 1 Introduction

Atherosclerosis is the underlying pathology of many cardiovascular diseases. Treatments for atherosclerosis such as vein grafting and stent implantation have a high failure rate due to restenosis [1], [2]. Restenosis is caused by intimal thickening as a result of inappropriate vascular smooth muscle cell (VSMC) activation, and superimposed atherosclerosis [3]. Current drug-eluting stents are coated with anti-proliferative treatments such as rapamycin, which dramatically reduce intimal thickening but result in higher thrombosis rates due to endothelial damage [2]. Furthermore, a clinically-proven pharmacological intervention for reduction of vein graft restenosis is not currently available. There is, therefore, still a need to identify novel treatments for intimal thickening (and thereby restenosis), which reduce VSMC migration and proliferation, whilst escaping detrimental effects on endothelial cell (EC) survival, migration and proliferation, as recently reviewed [4].

Homophilic cell–cell adhesions are mediated in VSMCs by cadherins, predominantly N-cadherin [5]. We have previously shown that cadherin-mediated cell–cell adhesion acts as a negative regulator of VSMC proliferation [5], [6], [7], [8]. Cadherin:catenin complexes are dismantled during proliferation in cultures of isolated human saphenous vein VSMCs [5] and after balloon injury of rat carotid arteries [8]. Dismantling of N-cadherin is mediated by MMP-dependent shedding from the cell surface, resulting in translocation of β-catenin to the nucleus [5] and up-regulation of cyclin D1 expression [7]. The role of N-cadherin in VSMC migration is less clear; one study showed that N-cadherin promotes migration [9], while another showed that N-cadherin inhibits migration [10]. N-cadherin can also affect the behaviour of endothelial cells [11], [12].

N-cadherin may also modulate VSMC proliferation and migration by interacting with the FGFR via a common HAV binding motif [13], [14]. We have previously observed that this interaction promoted VSMC survival [12]. Fibroblast growth factor (FGF) induces internalisation of FGF receptor (FGFR), followed by nuclear translocation, where FGFR acts as a general transcription regulator, activating the transcription of genes, such as FGF-2 and CREB, which are essential for proliferation [15]. E-cadherin is co-internalised with FGFR1 in human breast adenocarcinoma cells (MCF-7). Furthermore, overexpression of E-cadherin prevents the nuclear translocation of FGFR1 [16].

Soluble forms of cadherins, consisting only of the extracellular domain, can mimic the full-length form, inhibiting cell–cell adhesion but promoting responses that are normally stimulated by full length cadherins [11], [13]. Soluble N-cadherin also contains the HAV binding domain, enabling FGF-R binding. Soluble N-cadherin (SNC-Fc) is more suitable as a therapeutic than full length N-cadherin as it is smaller, and soluble and therefore more easily delivered. Soluble N-cadherin increased endothelial cell migration using the wounding assay and angiogenesis in the cornea [11], and we have previously shown that soluble N-cadherin significantly increased endothelial cell survival as well as VSMC survival [12].

In this study, we have investigated the effect of soluble N-cadherin on VSMC and endothelial cell proliferation and migration, and examined the signalling pathways involved. Additionally we have utilised a human ex-vivo organ culture model to investigate the effect of soluble N-cadherin on intimal thickening.

## 2 Materials and methods

### 2.1 Cell culture (VSMCs and HUVECs)

Human saphenous vein VSMCs at passage 2–8 were generated as described previously [5] from consenting patients undergoing coronary artery bypass surgery (Research Ethics Committee #04/Q2007/6, in accordance with the ethical standards laid down in the 1964 Declaration of Helsinki and its later amendments). Each experiment was performed with VSMCs from segments of vein from at least 3 different patients. Three separate preparations of human umbilical vein endothelial cells (HUVECs) were purchased from PromoCell and were cultured following the supplier's instructions.

### 2.2 Adenovirus production

Recombinant adenoviruses termed RAd FLNC, RAd SNC-Fc and RAd Fc (control) to over-express full length N-cadherin, the extracellular domain of N-cadherin conjugated to Fc or the Fc domain alone, respectively, were produced, as described previously [12]. The chimeric molecule comprised of SNC-Fc and mouse immunoglobulin G constant domain (with mutated Fc receptor and complement binding domains), enables protein A binding, as well as extending plasma half-life, and possibly increasing receptor–ligand interaction.

### 2.3 Mutation of SNC-Fc

Two fragments of SNC-Fc were cloned. The first contained the HAV and INPISGQ motifs, which are required for interaction with N-cadherin [17]. The second contained the IDPVNGQ motif, which is required for interaction with FGFR1 [17]. PCR mutagenesis was used to mutate the binding sites of SNC-Fc as previously described [18], and the mutated fragments were returned to SNC-Fc. Two plasmids were produced: “SNC-Fc N-Cadmut” and “SNC-Fc FGFRmut”.

### 2.4 Purification of Fc, SNC-Fc, and the mutated forms of SNC-Fc

CHO cells were infected with 50 pfu/cell of RAd Fc or RAd SNC-Fc, or alternatively transfected using Amaxa nucleofection with the mutated SNC-Fc plasmids following the manufacturer's instructions. The conditioned media was collected at 66 and 138 h after infection. Conditioned media was pooled and purification achieved with protein A columns (Amersham Biosciences). Protein concentrations were determined using the Bradford Protein assay (Sigma).

### 2.5 Assessment of proliferation

Proliferation in the presence of 20 ng/ml platelet-derived growth factor-BB (PDGF-BB) and 20 ng/ml basic fibroblast growth factor (bFGF) for VSMCs, or complete endothelial cell media for HUVECs, was assessed by bromodeoxyuridine (BrdU, Pubmed CID: 6035) incorporation by immunocytochemistry as previously [7], [19], and by immunocytochemistry for proliferating cell nuclear antigen (PCNA, see below)., VSMCs were subconfluent at the time of treatment. PDGF and bFGF were added at the same time as Fc or SNC-Fc to assess the effect of SNC-Fc on proliferation. In some experiments VSMCs were additionally treated with 30 μM SU5402 (Calbiochem) or siRNA (see below), to inhibit FGFR signalling, or transfected with FGFR1 (SP/NLS) to overexpress constitutively nuclear FGFR1. 150–200 cells were counted per condition to assess the percentage of proliferating cells.

### 2.6 Assessment of migration

The migration of VSMCs was determined by plating 4 × 10^4^ cells in 24 well plates with 10% (*v*/*v*) foetal calf serum and assessed by a scratch wound assay, as described previously [19]. Briefly, 4 h after plating, cells were pre-treated with Fc or SNC-Fc for 24 h then the monolayer of cells was scratched with a 1 ml pipette tip and treated with Fc or SNC-Fc and 2 μM hydroxyurea (Pubmed CID: 3657) for a further 18 h. The wound was photographed and the migrated distance calculating by subtracting the wound size at 18 h from the wound size at 0 h. Chemotaxis of VSMCs treated with Fc or SNC-Fc was determined using the Boyden chamber method, as described previously [20].

### 2.7 Immunocytochemistry

Cells were fixed with 3% paraformaldehyde (Pubmed CID: 712), permeabilised with 1% Triton X-100 (Pubmed CID 5590), blocked in 20% goat serum, incubated with FGFR antibody (R&D systems) diluted1:100 (5 μg/ml) or PCNA antibody from Abcam (ab18197), diluted 1:700 (1 μg/ml), followed by anti-mouse biotinylated secondary antibody (DAKO) or anti-rabbit biotinylated secondary antibody (DAKO), and Alexa fluor 488 anti-streptavidin (Life Technologies), and mounted in Prolong Gold with DAPI (Molecular Probes).

Dual immunocytochemistry for BrdU and β-galactosidase: briefly, cells were fixed with 3% paraformaldehyde, permeabilised with 1% Triton, treated with 2 N HCl, blocked in 20% goat serum, incubated with the BrdU (Sigma, 1:500) and β-galactosidase (Abcam, ab9361, 1:1000) primary antibodies, followed by anti-chicken biotinylated secondary antibody (DAKO), and Alexa fluor 488 anti-streptavidin and anti-mouse (Life Technologies), mounted without DAPI.

### 2.8 β-catenin signalling

TOPgal transgenic mouse VSMCs were grown from aortic explants, as described previously [7]. These VSMCs contain the β-galactosidase gene under the control of TCF responsive elements. TOPgal VSMCs were treated with 0.01% (*w*/*v*) trypsin, 1 mM CaCl_2_ (Pubmed CID 5284359)_,_ in phosphate-buffered saline (PBS) and seeded into a 24 well plate in the presence of SNC-Fc or Fc and 20 ng/ml PDGF-BB and 20 ng/ml bFGF to induce proliferation. β-galactosidase activity was quantified using the chemiluminescent Galactolight assay (Tropix) as described by the manufacturer's instructions. Further quantification was achieved using dual immunocytochemistry for BrdU and β-galactosidase (see above).

### 2.9 Western blot analysis and immunoprecipitation

SDS lysed cell extracts were subjected to Western blotting as described previously [5]. Blots were incubated overnight at 4 °C with primary antibodies diluted in 5% BSA/TBS. Antibodies were used at the following concentrations: N-cadherin (BD Biosciences: 610920, 1:2500), GAPDH (Chemicon: MAB374, 1:5000), and FGFR1 (R&D Systems: MAB658, 1:50). Bound antibodies were detected by rabbit anti-mouse or swine anti-rabbit horseradish peroxidase conjugated antibodies (Dako) and enhanced chemiluminescence (Amersham International). Stain free gel (BioRad) loading controls were used in some experiments. Fc tag was detected using the goat-anti-mouse secondary antibody (diluted 1:1000 in 5% Marvel in TBS-T).

Immunoprecipitation of GFP tag was performed as follows. FGFR1-GFP was overexpressed in full media using AMAXA (following the manufacturer's instructions) in 4 T25 flasks. After 24 h cells were treated with nothing (control), 200 pM Fc, SNC-Fc or SNC-Fc FGFR1mut in serum free media for 1 h. Cells were lysed in 400 μl RIPA buffer with complete protease inhibitor (Roche). Lysates were incubated on a rotator at 4 °C with GFP-agarose beads (abcam: ab69314) overnight, centrifuged and washed twice with RIPA buffer. The beads were resuspended in 30 μl Laemmli buffer (BioRad), and boiled at 95 °C for 5 min before loading onto a Western blot as described above. Fc tag detection was used.

### 2.10 FGFR1 siRNA knockdown

Two HP validated silencing RNA oligonucleotides (siRNA) for FGFR1 or control (Allstars Negative control siRNA) were purchased from Qiagen (catalogue numbers SI02224677, SI02224684 and 1027281). To confirm this, we additionally used siRNA oligonucleotides (FGFR and control) purchased from Santa Cruz (sc29316 and sc37007). VSMCs (8 × 10^5^) were subjected to Amaxa nucleofection with 250 pmol of FGFR1 or control siRNAs using the VSMC kit and U-25 programme following the manufacturer's instructions (Amaxa). VSMCs for proliferation analysis were seeded in serum free media and were treated with 100 pM Fc/SNC-Fc and proliferation induced with 20 ng/ml PDGF 24 h after nucleofection. Silencing was validated by quantitative PCR as described previously and below [6], [21], primers are shown in Table 1. Protein knockdown was assessed by Western blotting (as described above) and by immunocytochemistry for FGFR1 (see above).

### 2.11 Plasmid overexpression

GFP-tagged ORF clone of *Homo sapiens* fibroblast growth factor receptor 1 (FGFR1), transcript variant 3 as transfection-ready DNA was purchased from Insight Biotechnologies. VSMCs (8 × 10^5^) were subjected to Amaxa nucleofection with 5 μg of FGFR1 plasmid as outlined above. VSMCs were seeded in full media, then changed to serum free media after 18 h. Following 4 h in serum free media, VSMCs were treated with 20, 100 and 200 pM Fc/SNC-Fc in the presence of 20 ng/ml bFGF and 20 ng/ml PDGF. 4 h later, VSMCs were fixed and mounted in Prolong Gold with DAPI (Molecular Probes) and the percentage of cells with nuclear FGFR1 was calculated.

Constitutively nuclear FGFR1 (FGFR1 (SP/NLS)) was a kind gift from Professor Michal Stachowiak, University at Buffalo, New York City. VSMCs (8 × 10^5^) were subjected to Amaxa nucleofection with 5 μg of FGFR1 (SP/NLS) plasmid as outlined above. VSMCs were seeded in full media, then changed to serum free media after 18 h. Following 4 h in serum free media, VSMCs were treated with 200 pM Fc or SNC-Fc in the presence 20 ng/ml bFGF and 20 ng/ml PDGF. Proliferation was assessed by BrdU analysis after 24 h.

### 2.12 qPCR

Quiesced VSMCs were incubated with 20 ng/ml bFGF and 20 ng/ml PDGF in the presence of 200 pM Fc or SNC-Fc for 6 h and total RNA was extracted using miRNeasy kit (Qiagen) according to manufacturer's instructions. cDNA was derived using Transcriptor first strand cDNA synthesis kit (Roche) and subjected to quantitative PCR as described previously [6], [21] for cyclin D1, p21 and 18 s ribosomal RNA using primers (see Table 1) and Lightcycler 480 SYBR green I master (Roche) in accordance with manufacturer's instructions. Quantification of cyclin D1 and p21 messenger RNA (mRNA) was achieved after normalisation to 18 s ribosomal mRNA copies.

### 2.13 Human saphenous vein organ culture

Surplus segments of human saphenous vein (n = 5 different patients) were obtained from patients undergoing coronary artery bypass surgery and placed in organ culture as described previously [22]. This study was reviewed by the relevant ethics committee (REC number 04/Q2007/6), and therefore was performed in accordance with the ethical standards laid down in the 1964 Declaration of Helsinki. Endothelial denudation occurs during surgical preparation of vein [23], [24] consequently, we did not induce further endothelial denudation. The average endothelial coverage via QBend10 at time zero was 56.2 ± 8.7%. Segments of vein were cut open longitudinally then divided into 2 pieces crosswise, one was treated with 20 pM Fc and the other with 20 pM SNC-Fc and 10 μM BrdU and organ culture was performed as described previously [20]. Three micrometre paraffin-wax sections were stained with haematoxylin and eosin, and Miller's elastic van Gieson stain. Intimal thickness was quantified using image analysis (Image Pro-plus, MediaCybernectics, Wokingham, UK) by dividing the intimal area by the length of the vein segment, as described previously [22]. BrdU immunohistochemistry was performed as described previously [25]. VSMC migration was estimated by counting the number of cells in the intima that are not labelled with BrdU and dividing by the length of the vein segment. This method defines cells present within the intima that have arisen by migration alone, and not by migration and proliferation, as previously described [26]. ISEL was performed as described previously [27]. Immunohistochemistry for QBend10 was performed as described previously [22]. Dual QBend10 and ISEL immunohistochemistry was performed to quantify apoptosis in endothelial cells. ISEL was performed as previously [27] and then after using the avidin and biotin blocking kit (Vector) QBend10 staining was performed as previously [22] but using Extravidin alkaline phosphatase and Fast Red chromogen (Sigma).

### 2.14 Statistical analysis

Values are expressed as mean ± SEM. Data were analysed by ANOVA for multiple comparisons and the Student Newman Keuls post-test was used. For experiments with two groups, the paired t-test was utilised and the one-sample test was used for the endothelial cell coverage analysis. Differences were considered significant when p < 0.05.

The funding body was not involved in the interpretation of the data.

## 3 Results

### 3.1 Soluble N-cadherin reduced VSMC proliferation and migration

**Fig. 1:**
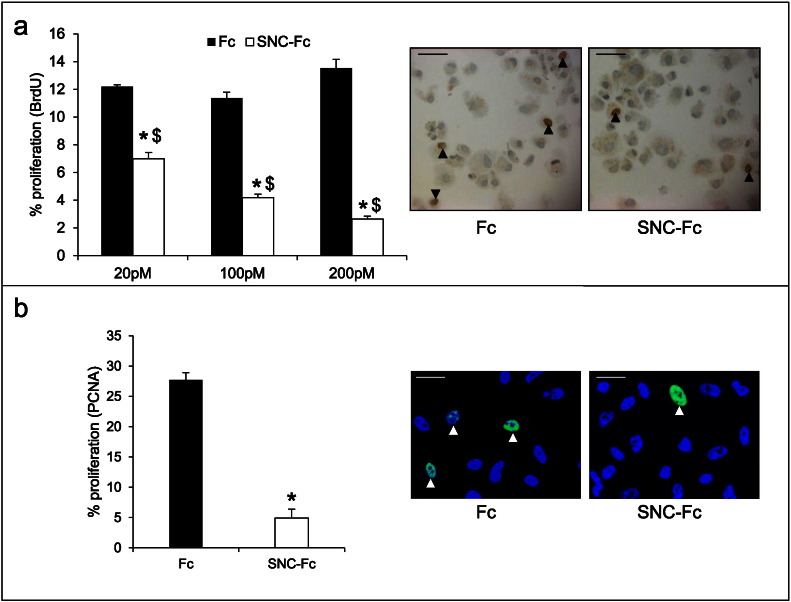
N-cadherin reduced VSMC proliferation. a: Proliferation (percentage of BrdU positive human VSMCs) following 24 h treatment with 20, 100 or 200 pM Fc or SNC-Fc in the presence of bFGF and PDGF. * indicates a significant difference from the Fc control, and $ indicates a significant difference from SNC-Fc at other doses, p < 0.05, n = 3. Representative images of immunocytochemistry for BrdU on VSMCs treated for 24 h with 200 pM Fc or SNC-Fc. Arrowheads indicate some positive nuclei (brown), negative nuclei are blue (haematoxylin). Scale bars represent 25 μm. b: Proliferation (percentage of PCNA positive human VSMCs) following 24 h treatment with 200 pM Fc or SNC-Fc in the presence of bFGF and PDGF. * indicates a significant difference from the Fc control, p < 0.05, n = 3. Arrowheads indicate some positive nuclei (green), negative nuclei are blue (DAPI). Scale bars represent 20 μm.

We observed that addition of 20–200 pM SNC-Fc significantly reduced VSMC proliferation in vitro (Fig. 1a), assessed by BrdU incorporation. This was confirmed with the 200 pM dose by immunocytochemistry for proliferating cell nuclear antigen (PCNA), a marker of proliferation (Fig. 1b). Addition of the same concentrations of SNC-Fc also significantly reduced VSMC migration, assessed by the wounding assay (Supplementary Fig. 1a), and this finding was confirmed with the 200 pM dose using the Transwell migration assay (Supplementary Fig. 1c). Although migration was significantly reduced, the degree of the effect of SNC-Fc on VSMC migration was small and was not dose-dependent (Supplementary Fig. 1a), even at doses ranging from 4 pM to 2000 pM (Supplementary Fig. 1b). Therefore, we focused all further experiments on the effects of SNC-Fc on proliferation.

### 3.2 Soluble N-cadherin did not affect N-cadherin levels or β-catenin signalling

**Fig. 2:**
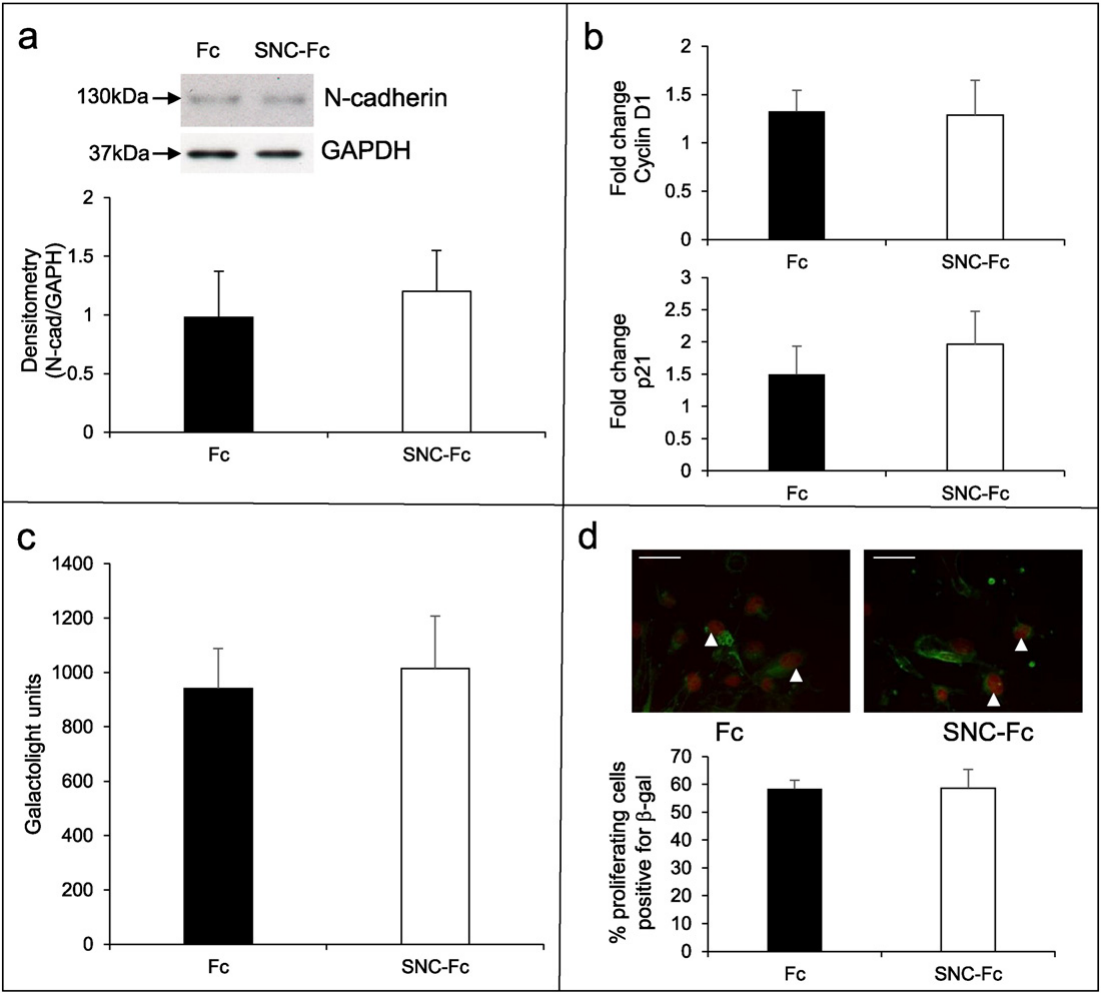
SNC-Fc had no effect on β-catenin signalling. a: Representative Western blots of N-cadherin and GAPDH proteins following 24 h treatment with 200 pM Fc or SNC-Fc in the presence of bFGF and PDGF. Chart shows densitometry analysis of N-cadherin normalised by GAPDH, n = 3. b: qPCR analysis of β-catenin target genes, cyclin D1 and p21 levels following 6 h treatment with 200 pM Fc or SNC-Fc in the presence of bFGF and PDGF, normalised to 18S, n = 3. c: Galactolight assay (to measure β-galactosidase activity, and thereby β-catenin signalling) measured in arbitrary units in TOPgal VSMCs treated with 200 pM Fc or SNC-Fc in the presence of bFGF and PDGF, n = 3. d: Representative images of dual immunocytochemistry for β-galactosidase (green) and BrdU (red) on TOPgal VSMCs treated with 200 pM Fc or SNC-Fc in the presence of bFGF and PDGF. Chart shows the percentage of proliferating cells (BrdU positive) that were positive for β-galactosidase, n = 3. Arrowheads indicate some positive cells (red nuclei and green cytoplasm). Scale bars represent 25 μm.

We have previously demonstrated that N-cadherin levels are reduced and β-catenin signalling is increased in proliferating VSMCs [5]. Consequently, we examined whether N-cadherin levels and β-catenin signalling were affected by treatment with SNC-Fc compared to Fc to determine whether this contributed to the inhibition of proliferation. Interestingly, SNC-Fc had no significant effect on the levels of full length N-cadherin in human VSMCs after stimulation of proliferation with PDGF-BB and bFGF (Fig. 2a). Additionally, SNC-Fc treatment had no effect on the mRNA levels of the β-catenin target genes, cyclin D1 and p21 (Fig. 2b) in human VSMCs.

To investigate the effect of SNC-Fc on TCF-dependent β-catenin signalling directly, we used VSMCs isolated from the aortas of TOPgal transgenic mice. These mice have the β-galactosidase gene under the control of TCF responsive elements so β-catenin/TCF signalling can be assessed by quantification of β-galactosidase [7], [21]. We detected a significant increase in β-catenin signalling in proliferating cells compared to quiescent cells, as assessed by immunocytochemistry for β-galactosidase (45.4 ± 6.3 vs. 18.0 ± 5.1, % cells positive for β-galactosidase) and by the galactolight luminescent assay for β-galactosidase activity. However, SNC-Fc treatment during PDGF-BB and bFGF induced proliferation had no effect on β-catenin signalling, detected by the galactolight luminescent assay for β-galactosidase activity and by immunocytochemistry for β-galactosidase (Fig. 2c and d, respectively).These data show that under these experimental conditions, the mechanism by which SNC-Fc reduces proliferation is not through inhibition of TCF dependent β-catenin signalling or by altering full length N-cadherin protein levels.

### 3.3 FGFR was essential for the anti-proliferative effect of soluble N-cadherin

Due to the lack of effect of SNC-Fc on β-catenin signalling and N-cadherin levels we investigated an alternative mechanism. We focused on FGFR signalling as we have previously shown that SNC-Fc can activate FGFR and reduce apoptosis [12].

**Fig. 3:**
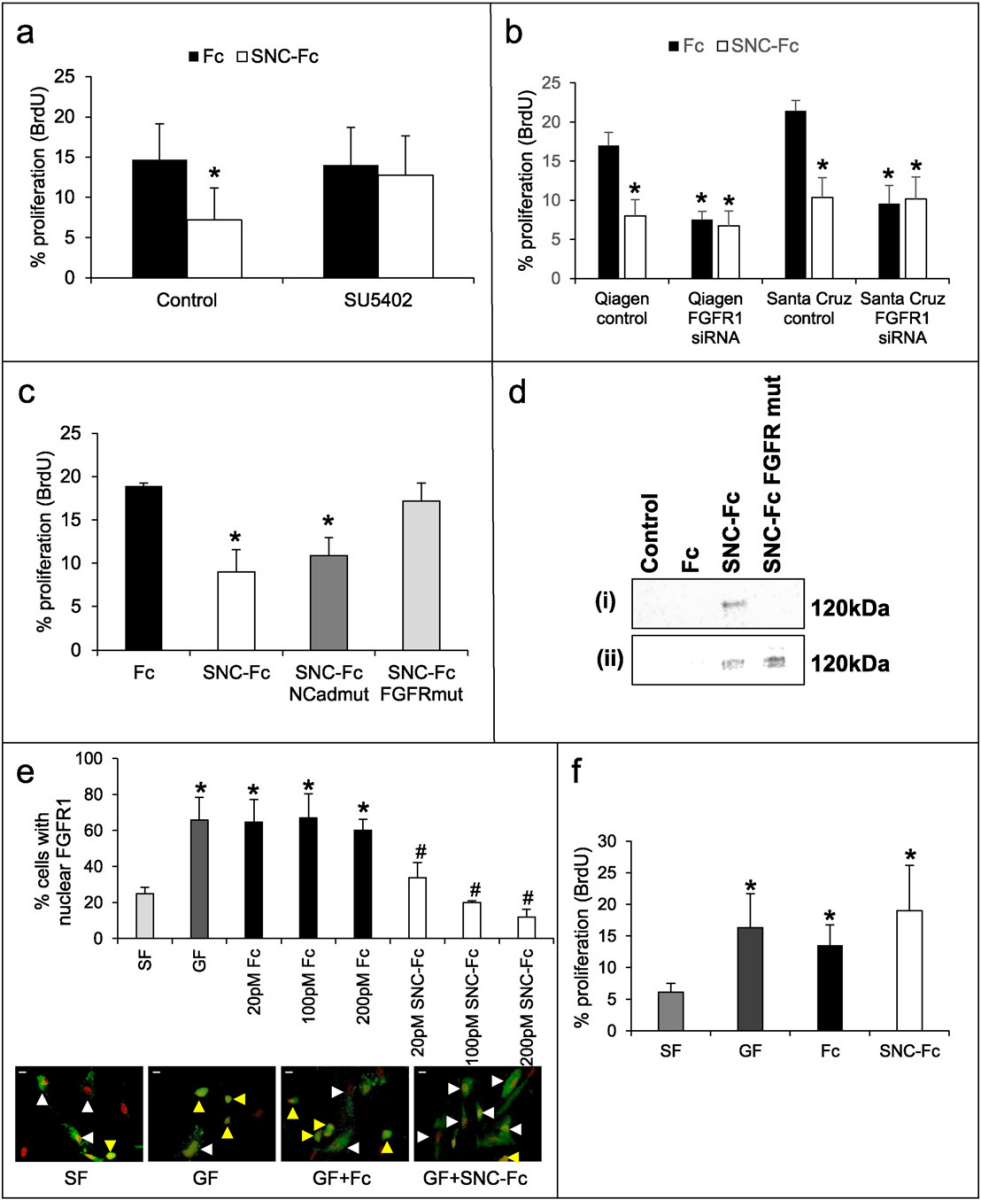
FGF-R was essential for the anti-proliferative effect of soluble N-cadherin. a: Proliferation (percentage of BrdU positive human VSMCs) following treatment with SU5402 (an inhibitor of FGFR1) and 100 pM Fc or SNC-Fc, in the presence of bFGF and PDGF for 24 h. * indicates a significant difference from the Fc control, n = 3. b: Proliferation (percentage of BrdU positive human VSMCs) following siRNA for FGFR1 and treatment with 100 pM Fc or SNC-Fc in the presence of bFGF and PDGF for 24 h. * indicates a significant difference from the relevant Fc control, n = 3. c: Proliferation (percentage of BrdU positive human VSMCs) following 24 h incubation with bFGF and PDGF and 100 pM Fc, SNC-Fc or SNC-Fc with either the N-cadherin binding site mutation (SNC-Fc N-cadmut) or FGF-R binding site mutated (SNC-Fc FGFRmut). * indicates a significant difference from the Fc control, n = 3. d: Western blot for Fc-tag in VSMCs overexpressing FGFR1GFP and incubated with Fc/SNC-Fc/SNC-Fc for 1 h, before immunoprecipitation for GFP tagged FGFR and Western blotting (i). Western blot for Fc-tag in VSMCs overexpressing full length N-cadherin and incubated with Fc/SNC-Fc/SNC-Fc FGFR for 1 h, before lysis and Western blotting (ii). e: The percentage of cells with nuclear FGFR following AMAXA transfection with FGFR1 plasmid and treatment with either serum free media (SF), bFGF and PDGF (GF), and 20, 100 or 200 pM Fc or SNC-Fc plus GF. * indicates a significant difference from SF control, # indicates a significant difference from GF and Fc plus GF, n = 3. Representative images to show nuclear translocation of FGFR1 for the 200 pM dose. Scale bars represent 10 mm. Yellow arrowheads indicate cells with nuclear FGFR1 (green), white arrowheads indicate cells with cytoplasmic FGFR1. Nuclei have been psuedocoloured in red. f: Proliferation (percentage of BrdU positive human VSMCs) following transfection with FGFR1(SP/NLS), a constitutively nuclear form of FGFR1, and treatment with 200 pM Fc or SNC-Fc in the presence of bFGF and PDGF for 24 h. * indicates a significant difference from SF, n = 3.

Inhibition of FGFR using a synthetic inhibitor (SU5402) ablated the anti-proliferative effect of SNC-Fc (Fig. 3a). This was confirmed using two different siRNA sequences for FGFR1 (the predominant FGFR in human VSMCs, Fig. 3b). FGFR1 mRNA and protein levels were significantly reduced following siRNA treatment (Supplementary Fig. 1). This suggested that FGFR was required for the anti-proliferative effect of SNC-Fc. Unsurprisingly, FGFR1 siRNA itself significantly reduced VSMC proliferation.

Further support for this was gained by using mutated versions of SNC-Fc, which prevent interactions with either N-cadherin or FGFR [17]. Proliferation, as assessed by BrdU incorporation, was still reduced by SNC-Fc when the N-cadherin binding site was mutated (SNC-Fc N-Cadmut), suggesting that N-cadherin binding is not necessary for the anti-proliferative effect of SNC-Fc (Fig. 3c). However, when the FGFR binding site was mutated (SNC-Fc FGFRmut), proliferation could no longer be reduced (Fig. 3c). To prove that the mutation prevents the interaction of SNC-Fc with the FGFR, we have performed an immunoprecipitation. FGFR1-GFP was overexpressed, and cells were treated with Fc/SNC-Fc or SNC-Fc FGFRmut. Immunoprecipitation for GFP was used to pull down FGFR1-GFP. Western blotting for the Fc tag was performed, to show that only SNC-Fc, but not Fc nor SNC-Fc FGFRmut were bound to the FGFR1-GFP (Fig. 3d (i)). To prove that SNC-Fc FGFR1 remains otherwise functional, we have shown that it can still bind to the full length N-cadherin molecule (Fig. 3d (ii)). Full length N-cadherin was overexpressed using RAd FLNC, as previously described [28]. Cells were then incubated with 200 pM Fc/SNC-Fc or SNC-Fc FGFRmut for 1 h, then lysed and Western blotting performed for the Fc tag. As shown in Fig. 3d (ii), whilst Fc has not bound to the cells, and therefore is not present in the cell lysate, both SNC-Fc and SNC-Fc FGFRmut have bound, suggesting that functionality has been retained.

To investigate the mechanism by which SNC-Fc reduced proliferation by binding to the FGFR, we looked at the effect of SNC-Fc on the nuclear localisation of FGFR. AMAXA nucleofection was utilised to transfect human saphenous vein VSMCs with GFP labelled FGFR1 (transfection efficiency was 36.7 ± 3.3%), prior to treatment with PDGF and bFGF and 20, 100 and 200 pM Fc or SNC-Fc. As observed by Bryant et al. [16], we found that addition of bFGF and PDGF caused nuclear translocation of the FGFR1 (Fig. 3e). Nuclear translocation was prevented by addition of SNC-Fc, and this occurred in a dose responsive manner (Fig. 3e). FGFR1 (SP/NLS) causes FGFR1 to be constitutively nuclear. We utilised this plasmid to show that when FGFR1 is constitutively nuclear, SNC-Fc cannot reduce VSMC proliferation (Fig. 3f), providing causal evidence that FGFR1 nuclear translocation is the mechanism by which SNC-Fc reduces VSMC proliferation.

### 3.4 Soluble N-cadherin reduced intimal thickening

**Fig. 4:**
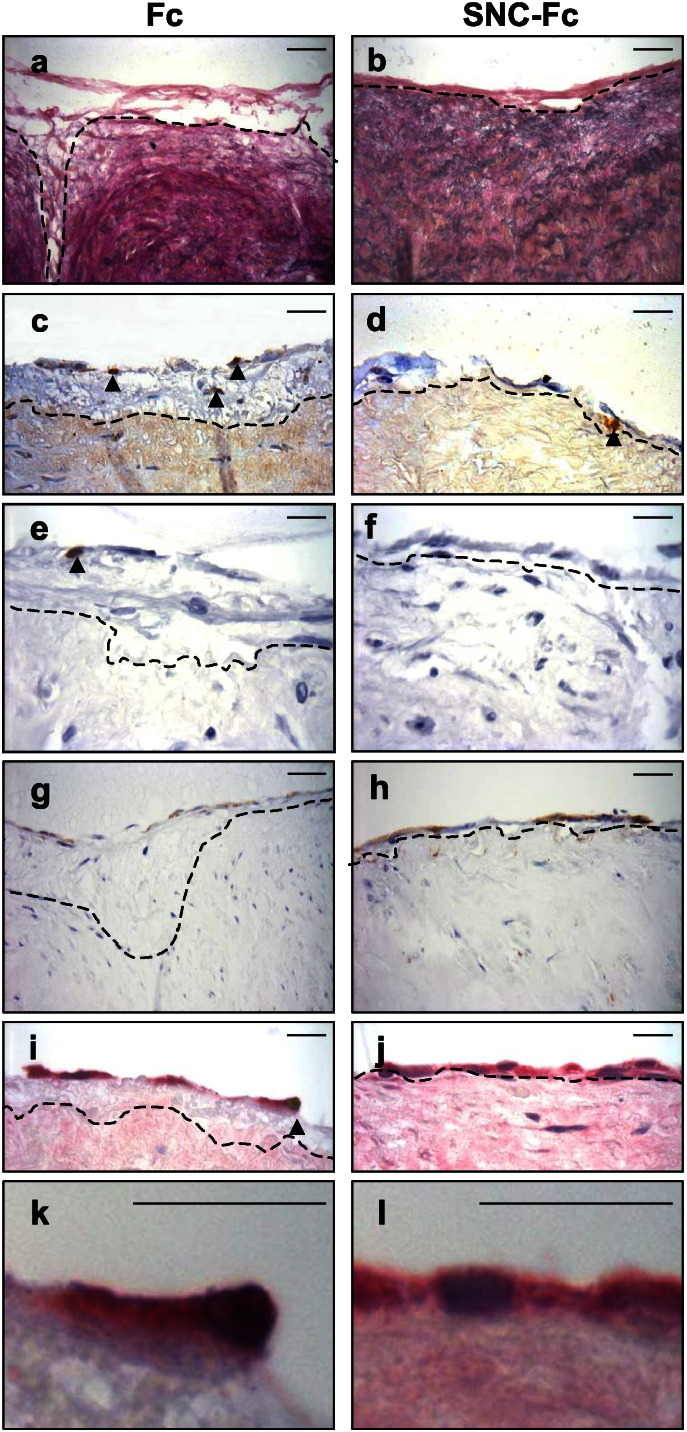
SNC-Fc reduced intimal thickening in an *ex vivo* organ culture model. Representative images of: a and b: Elastin van Gieson staining to show intimal size. c and d: Immunohistochemistry for BrdU incorporation (brown nuclei: proliferation). e and f: ISEL (brown nuclei: apoptosis). g and h: Immunohistochemistry for QBend10 (brown colour: endothelial cells). i and j: Dual immunohistochemistry for QBend10 (pink, endothelial cells) and ISEL (brown nuclei, apoptosis). k and l: Higher power images of the dual QBend10 (pink) and ISEL (brown) to clearly show the double staining in i. Dotted line delineates the intimal:medial boundary. Arrowheads indicate some of the positive cells. Scale bars in a-d and g and h represent 20 μm, scale bars in e, f, i, j, k and l represent 40 μm, n = 5.

As a result of the beneficial effects on VSMC proliferation we aimed to determine whether SNC-Fc could suppress intimal thickening. The effect of SNC-Fc on intimal thickening was investigated using a well-validated human ex vivo organ culture model [29], [30], [31], [32]. Segments of human saphenous vein were incubated in culture media containing 20 pM Fc or SNC-Fc for a period of 14 days. During this time an intima forms on the lumenal surface of the vein due to proliferation and migration of VSMCs [20]. SNC-Fc significantly reduced intimal area (Fig. 4a–b, Table 2) predominantly through inhibition of intimal cell proliferation, as measured by the percentage of cells positive for BrdU incorporation (Fig. 4c–d, Table 2). SNC-Fc also significantly reduced the percentage of cells positive for ISEL, a marker of apoptosis (Fig. 4 e–f, Table 2). However, there was no effect on medial density or migration of cells into the intima (indirectly measured as the number of non-BrdU labelled cells in the intima, Table 2). Importantly, SNC-Fc significantly increased endothelial coverage. Endothelial coverage was measured as the length of vein positive for QBend10 divided by the total length and expressed as a percent of the Fc control (Fig. 4g–h). Endothelial coverage between the two groups was comparable at the start of the study, we found that segment to segment reproducibility was high (coefficient of variation = 0.06 ± 0.01). Dual immunohistochemistry for apoptosis (ISEL) and endothelial cells (Q-Bend10) showed that SNC-Fc treatment significantly reduced endothelial cell apoptosis (Fig. 4i–l, Table 2), which agrees with our previous in vitro data [12].

### 3.5 Soluble N-cadherin had no effect on endothelial cell proliferation and migration

**Fig. 5:**
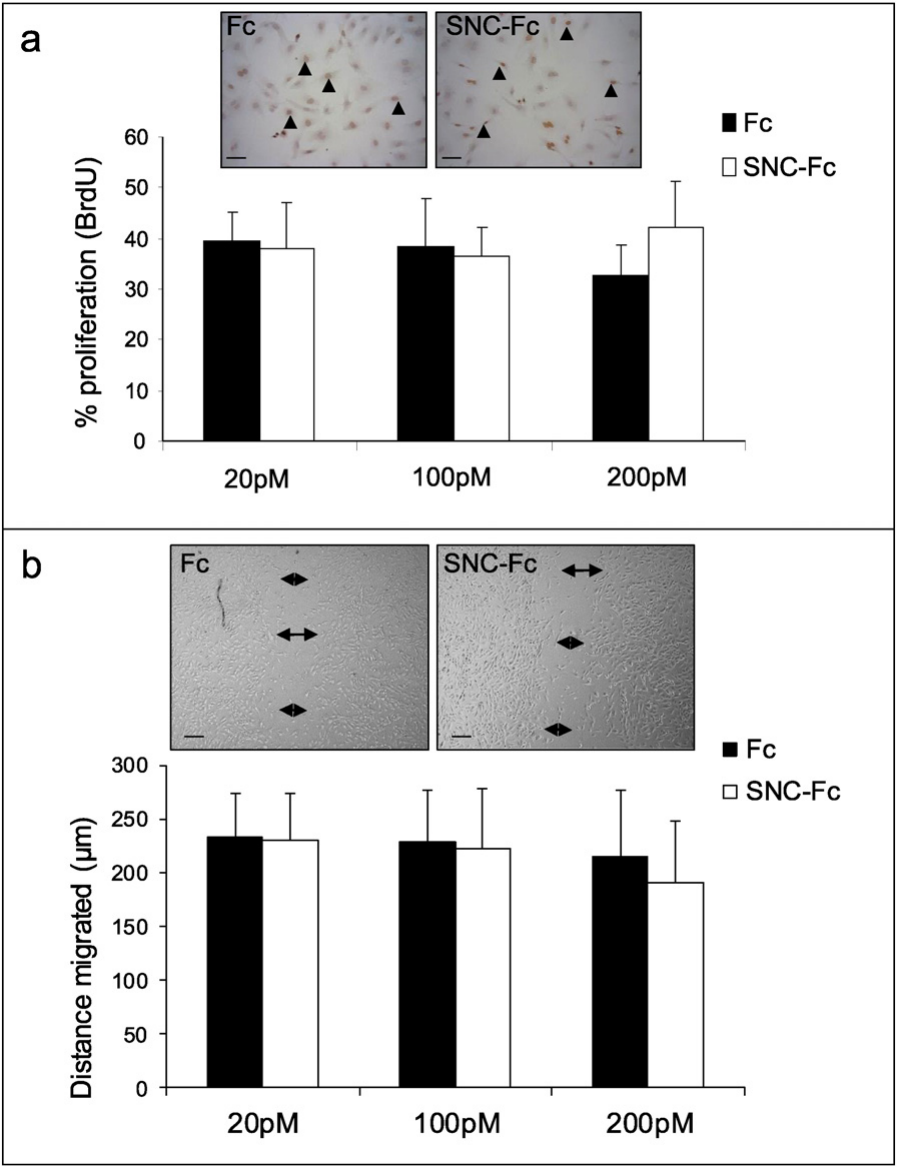
SNC-Fc had no effect HUVEC proliferation and migration. a: Proliferation (percentage of BrdU positive HUVECs) following 24 h incubation with 20, 100 or 200 pM Fc or SNC-Fc, in the presence of 2% FCS, n = 3. Arrowheads indicate BrdU positive cells (brown), negative cells have blue nuclei (haematoxylin). Scale bars represent 20 μm. b: Distance migrated in μm after 18 h (scratch wound assay) by HUVECs treated with 20, 100 or 200 pM Fc or SNC-Fc, in the presence of 2% FCS, n = 3. Scale bars represent 100 μm. Arrows indicate the wound edges.

Endothelial cell coverage is reliant on endothelial cell survival, migration and proliferation. We have previously published that SNC-Fc increases endothelial cell survival [12], which therefore contributes to the increased endothelial coverage. We also examined the effect of SNC-Fc on endothelial cell (HUVEC) proliferation and migration. However, 20–200 pM of SNC-Fc did not reduce endothelial cell proliferation (as assessed by BrdU incorporation) or migration (as assessed by the scratch wound assay) compared to Fc control (Fig. 5 a and b).

## 4 Discussion

VSMC proliferation and migration are major contributors to intimal thickening and restenosis after clinical interventions for atherosclerosis such as stenting and vein grafting, and cause high failure rates of these interventions [2], [33]. Consequently, anti-proliferative and anti-migratory strategies are highly sought after for improving vein graft patency and for use in drug-eluting stents. The major problem with the currently available anti-proliferative treatments is that they have a detrimental effect on re-endothelialisation. Therefore, a drug which reduces VSMC proliferation and/or migration, but has no effect, or increases endothelial cell proliferation, migration and/or survival is desirable. In this study, we found that SNC-Fc significantly reduced VSMC proliferation, and to a lesser extent migration, whilst having no effect on HUVEC proliferation and migration in vitro. Using a human saphenous vein ex vivo organ culture model of intimal thickening, we showed that SNC-Fc significantly reduced intimal area, whilst reducing endothelial cell apoptosis and thereby increasing endothelial coverage. This study suggests that SNC-Fc may have potential as a therapeutic for restenosis.

We have previously demonstrated that full length N-cadherin inhibits VSMC proliferation [7], and that cadherin:catenin complexes are dismantled during proliferation in cultures of isolated human saphenous vein VSMCs [5]. In this study we have also shown that SNC-Fc, a potentially useful therapeutic soluble molecule, significantly inhibited VSMC proliferation. This corroborates a previous observation that a soluble VE-cadherin retarded endothelial cell proliferation [34]. In contrast, our study revealed that SNC-Fc had no significant effect on HUVEC migration or proliferation. Previous studies in endothelial cells have shown that SNC-Fc increased endothelial cell migration using the wounding assay and angiogenesis in the cornea [11]. The discrepancy may be the result of cell type and species differences, or since we utilised considerably lower doses (2–200 pM) of SNC-Fc compared to the previous study (56–112 nM). A recent study in isolated cells has suggested that atorvastatin (but not other statins) more potently reduced VSMC proliferation than EC [35], but our study identifies SNC as an agent that has no effect on EC proliferation whilst potently reducing VSMC proliferation. Moreover, we illustrate using our organ culture model enhance EC coverage of vein segments as a result of SNC treatment.

N-cadherin mediates directed cell migration by increasing cell-cell adhesion in various cell types (for example [36], [37]). We have also shown that full length N-cadherin also significantly increased cell-cell contacts in VSMCs [7]. SNC-Fc has the opposite effect, and significantly reduces both the ability of single VSMCs to aggregate and form cell–cell adhesions [12], indicating that SNC-Fc acts as a mimetic interfering with cell-cell association. As cell–cell contacts are important for directed migration, this provides a potential mechanism for how SNC-Fc significantly reduced VSMC migration. However, as seen in Supplementary Fig. 1, the effect on migration is limited, and not as large as for proliferation.

We have used both bFGF and PDGF to induce proliferation in the in vitro experiments. Due to the fact that PDGF results in cellular release of endogenous bFGF [38], using these growth factors individually was not useful in teasing out any specific effects of bFGF compared to PDGF.

When we investigated the mechanism by which SNC-Fc reduced VSMC proliferation, we anticipated that SNC-Fc would act as a mimetic, binding full length N-cadherin at the cell membrane and making the cell believe it is participating in a cell–cell junction, and therefore retarding proliferation. We predicted that this would result in accumulation of N-cadherin and β-catenin at the cell membrane. However, under these experimental conditions, we saw no change in the levels of N-cadherin in the cell, and no change in TCF dependent β-catenin signalling, nor in the levels of β-catenin target genes (cyclin D1 and p21). We have previously shown that N-cadherin is higher in quiescent VSMC than in proliferating VSMCs [5], suggesting that it is lost during proliferation. However, we did not observe this change in N-cadherin levels in these experiments, suggesting that SNC-Fc does not interfere with N-cadherin levels. This was unexpected, as N-cadherin and β-catenin play pivotal roles in VSMC proliferation.

Consequently, we examined an alternative mechanism involving the FGFR. N-cadherin shares an HAV binding motif and can interact with the FGFR [13], [14], [39], [40], and we have previously shown that SNC-Fc can activate FGFR signalling [12]. Therefore, we investigated whether FGFR1, the predominant FGF receptor expressed by human saphenous VSMCs, was involved in the anti-proliferative effect of SNC-Fc. Firstly, we used a chemical inhibitor of FGFR, SU5402, which prevented the anti-proliferative effect of SNC-Fc. At this dose, SU5402, is shown to be specific for the FGFR and have no inhibitory effect on the PDGF receptor. We confirmed this using two separate siRNA sequences, which showed the same result: that when FGFR is knocked down, SNC-Fc can no longer reduce proliferation. Unfortunately, but understandably, siRNA knockdown of FGFR1 caused a reduction of proliferation. This complicates the results, as it is possible that SNC-Fc is just not able to reduce proliferation any further than it has been already. However, we have seen levels of proliferation lower than ~ 5% when we quiesce the cells with serum free medium, suggesting that it is possible. We believe that combined with the rest of the data in Fig. 3, it is compelling that the FGFR1 is involved in this mechanism.

To provide additional evidence for the interaction of SNC-Fc and FGFR1, we created mutations which prevented binding to either the FGFR or N-cadherin. We found that when the N-cadherin binding site was mutated, this form of SNC-Fc (SNC-Fc N-cadmut) could still reduce proliferation. However, when the FGFR binding site was mutated, this form of SNC-Fc (SNC-Fc FGFRmut) could no longer reduce VSMC proliferation, suggesting that this interaction is vital for the anti-proliferative effect of SNC-Fc. Additionally, we have shown direct evidence, by means of an immunoprecipitation, that SNC-Fc and the FGFR can interact, and that this interaction is ablated by the mutation of the FGFR binding site. We can also show that SNC-Fc FGFRmut can still bind to N-cadherin on the cell surface, indicating that the mutation has not disrupted other functions of SNC-Fc, nor its tertiary protein structure.

It appeared counterintuitive to us that SNC-Fc could be binding to the FGF-R and reducing proliferation. Especially so, as we have previously published that when bound to the FGF-R, SNC-Fc can activate PI-3 kinase and Akt signalling, resulting in increased cell survival [12]. This is a prime example of how different cellular situations and the presence of different factors in the cellular milieu can affect the signalling outcome.

It is known that when FGF binds to the FGFR, they are co-internalised and the complex translocates to the nucleus, where FGFR acts as a general transcription regulator, activating the transcription of genes, such as FGF-2 and CREB, which are essential for proliferation [15]. We postulated that binding of SNC-Fc to FGFR1 (in the presence of bFGF) may prevent the internalisation and nuclear translocation of FGFR, and thus reduce proliferation. Using a GFP labelled FGFR1, we confirmed that in the presence of bFGF, FGFR1 is translocated to the nucleus [15], [16]. Furthermore, we showed that this translocation was prevented by the addition of SNC-Fc. The percentage of cells with nuclear FGFR1 was SNC-Fc dose dependent, as was the inhibition of proliferation, suggesting that these events are linked. We also showed that when FGFR1 is constitutively nuclear, SNC-Fc cannot reduce VSMC proliferation, providing further evidence that SNC-Fc reduces proliferation by preventing nuclear translocation of FGFR1.

The identification of the FGFR binding site alone as the active site is extremely advantageous as it indicates the possibility of designing novel specific peptides which can be evaluated as a smaller therapeutic in the future.

The ex vivo human saphenous vein organ culture model of intimal thickening is an extremely useful, robust and relevant model of intimal thickening [20], [27]. Importantly we showed that SNC-Fc reduced intimal thickening by approximately 85% in this model. This appeared to be the result of retarded proliferation in the media and intima. There was however, no effect on medial density, which is reassuring as reducing medial density could result in thinning and weakening of the graft. SNC-Fc also reduced apoptosis in the media and the intima, which at first may appear counter-intuitive, as one would predict that reducing apoptosis would increase intimal cell number. However, the rates of apoptosis in this model are relatively low and therefore may have only a small impact on total cell number. In addition, when we performed dual immunohistochemistry for endothelial cells and ISEL, it was clear that many of the apoptotic intimal cells were endothelial cells. SNC-Fc inhibited endothelial cell apoptosis in the organ culture model in a similar manner as we previously observed in vitro [12], which presumably contributes to the increased endothelial coverage. Additionally, the anti-apoptotic effect of SNC-Fc on endothelial cells could indirectly reduce VSMC proliferation. Vascular endothelial cells constitutively express endothelial nitric oxide synthase, resulting in production of nitric oxide, which inhibits proliferation of VSMCs [41]. Consequently, the enhanced endothelial coverage will result in higher levels of nitric oxide and result in down-regulation of VSMC proliferation.

In summary, this study highlights SNC-Fc as a potential therapeutic agent for late vein graft failure and also in-stent restenosis, as it retards intimal thickening via beneficial actions on VSMCs and endothelial cells. We showed for the first time that SNC-Fc significantly reduced intimal cell proliferation, whilst having no detrimental effect on endothelial cell proliferation and migration. Importantly, we demonstrate SNC-Fc significantly reduced intimal area, and increased endothelial coverage in the organ culture model of intimal thickening. Moreover, the identification of the active site of SNC-Fc could lead to production of a smaller clinically useful peptide, which may be used as a stent coating or delivered directly to the vein graft.

## Conflicts of interest

The authors declare that they have no conflict of interest.

## Figures and Tables

**Table 1:** QPCR primers.

Primer name	Sequence/catalogue number	Company
p21	F: 5′-AGGATCCATGTCAGAACCGGCTGG-3′ R: 5′-CAGGATCCTGTGGGCGGATTAGGG-3′	Sigma
Cyclin D1	F: 5′-AGTAGCAGCGAGCAGCAGAGT-3′ R: 5′-TTCATCTTAGAGGCCACGAA-3′	Sigma
18S	F:5′-CGCGGTTCTATTTTGTTGGT-3′ R: 5′-CTTCAAACCTCCGACTTTCG-3’	Sigma
FGFR1	QT00102837	Qiagen

**Table 2:** Effect of SNC-Fc on human saphenous vein organ culture model.

	Fc	SNC-Fc
Intimal area/length of vein segment (μm)	8.68 ± 1.85	1.28 ± 0.60⁎
% intimal cells BrdU positive	30.4 ± 7.2	13.6 ± 3.6⁎
% medial cells BrdU positive	11.6 ± 3.1	5.0 ± 1.3⁎
Medial density (cell/m^2^ × 1000)	14.13 ± 3.3	15.44 ± 2.8
Migration into intima (non-BrdU cells/length × 1000)	2.9 ± 2.0	2.5 ± 0.8
% intimal cells ISEL positive	6.23 ± 1.6	3.06 ± 0.73⁎
% medial cells ISEL positive	3.18 ± 0.60	1.95 ± 0.22⁎
Endothelial coverage (fold change from Fc)	1 ± 0	2.92 ± 0.68⁎
% endothelial cells ISEL positive	9.44 ± 1.58	3.49 ± 1.0⁎

Mean ± SEM.

⁎ Indicates a significant difference from the Fc control sample, paired Student's t-test, p < 0.05, n = 5.
