# Supplementary material for: Soluble N-cadherin: A novel inhibitor of VSMC proliferation and intimal thickening
Source: Vascul Pharmacol. 2016 Mar;78:53–62. doi: 10.1016/j.vph.2015.11.040 (PMC4749540; doi:10.1016/j.vph.2015.11.040)
Supplement: Supplementary file 1 — Supplementary figures [file mmc1.pdf]

Supplementary Figure 1:

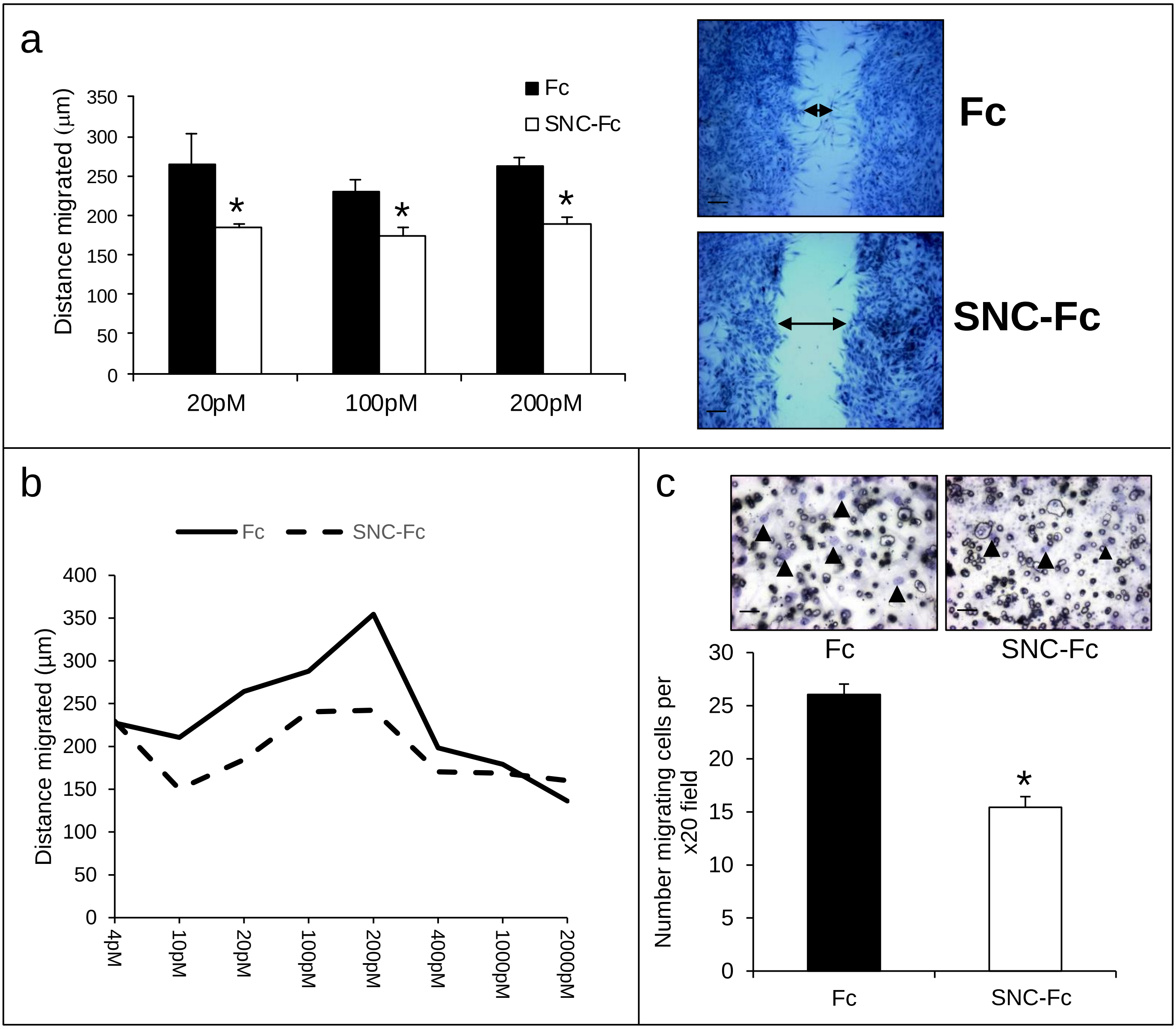

Supplementary Fig 1: <u>SNC modestly reduced VSMC migration.</u>

a: Migration: quantification of distance migrated by human VSMCs in the wounding assay 18 hours after treatment with 20, 100 or 200pM Fc or SNC-Fc in the presence of bFGF and PDGF. * indicates a significant difference from the Fc control, $p<0.05$, n=4. Representative images of the wounding assay. Arrows indicate wound edges. Scale bar represents 100µm.

c: Quantification of the distance migrated by human VSMCs in the wounding assay 18 hours after treatment with 4 - 2000pM Fc or SNC-Fc, in the presence of bFGF and PDGF, n=1.

d: Migration: quantification of the number of migrating cells / field in the Transwell assay after treatment with 200pM Fc or SNC-Fc, in the presence of bFGF and PDGF. * indicates a significant difference from the Fc control, n=4. Representative images of tranwells, arrowheads indicate migrated cells. Scale bars represent 10µm.

Supplementary Figure 2:

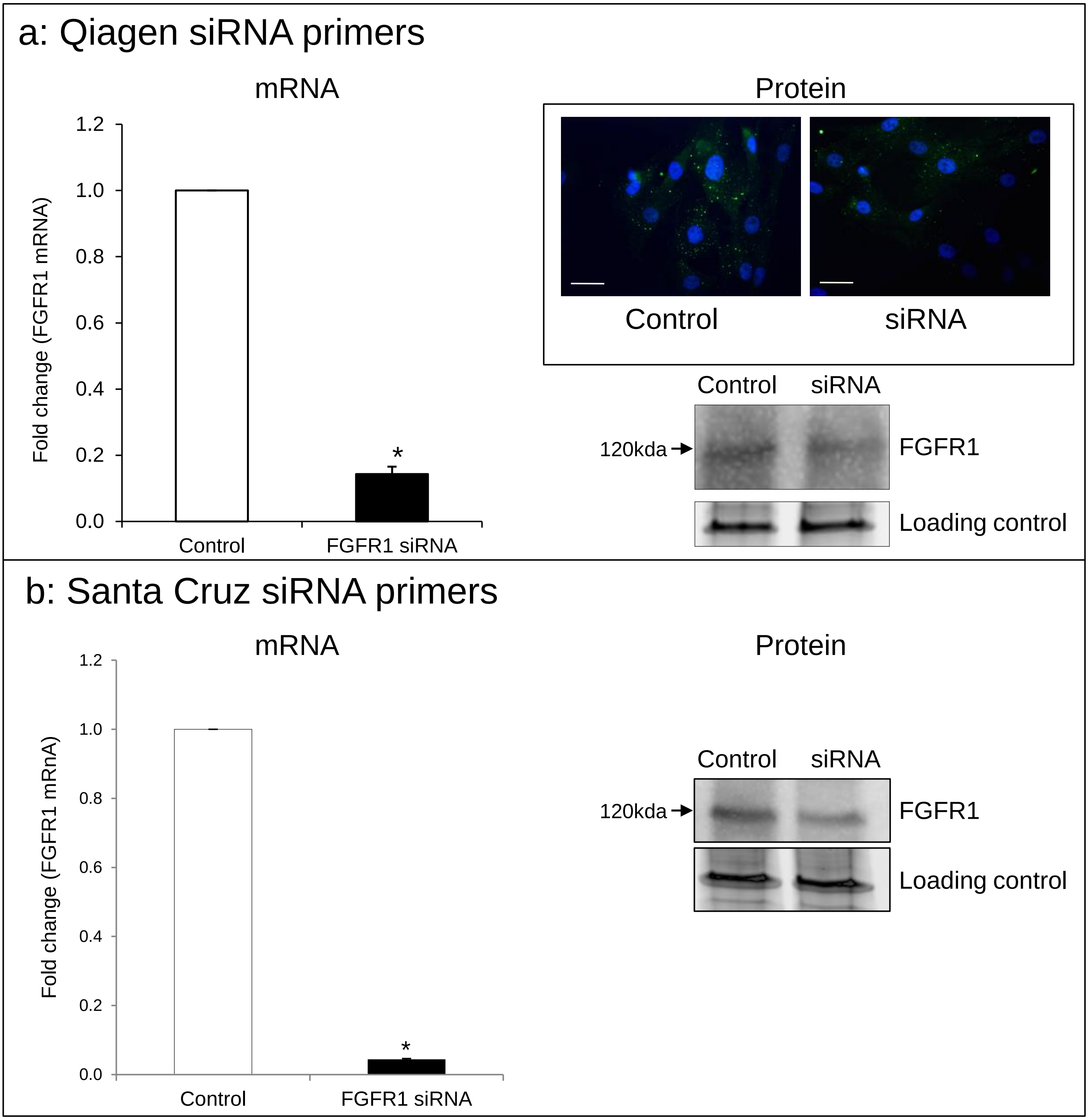

Supplementary Fig 1: Knockdown of FGFR1 using siRNA.
a: Knockdown of FGFR1 mRNA following FGFR1 siRNA with the Qiagen primers. * indicates a significant difference from the Fc control, n=3. Representative images of immunocytochemistry for FGFR1 (green) following siRNA treatment, nuclei blue (DAPI). Scale bar represents 20μm. Representative Western blot of FGFR1 protein.
b: Knockdown of FGFR1 mRNA following FGFR1 siRNA with the Santa Cruz primers. * indicates a significant difference from the Fc control, n=3. Representative Western blot of FGFR1 protein.
